# Transgressive Potential Prediction and Optimal Cross Design of Seed Protein Content in the Northeast China Soybean Population Based on Full Exploration of the QTL-Allele System

**DOI:** 10.3389/fpls.2022.896549

**Published:** 2022-07-12

**Authors:** Weidan Feng, Lianshun Fu, Mengmeng Fu, Ziqian Sang, Yanping Wang, Lei Wang, Haixiang Ren, Weiguang Du, Xiaoshuai Hao, Lei Sun, Jiaoping Zhang, Wubin Wang, Guangnan Xing, Jianbo He, Junyi Gai

**Affiliations:** ^1^Soybean Research Institute/MARA National Center for Soybean Improvement/MARA Key Laboratory of Biology and Genetic Improvement of Soybean (General), Nanjing Agricultural University, Nanjing, China; ^2^State Key Laboratory for Crop Genetics and Germplasm Enhancement, Nanjing Agricultural University, Nanjing, China; ^3^Tieling Academy of Agricultural Sciences, Tieling, China; ^4^Mudanjiang Research and Development Center for Soybean/Mudanjiang Experiment Station of the National Center for Soybean Improvement, Mudanjiang Branch of Heilongjiang Academy of Agricultural Sciences, Mudanjiang, China; ^5^Jiangsu Collaborative Innovation Center for Modern Crop Production, Nanjing Agricultural University, Nanjing, China

**Keywords:** Northeast China soybean germplasm population (NECSGP), seed protein content (SPC), restricted two stage multi-locus model GWAS (RTM-GWAS), QTL-allele matrix, optimal cross prediction, transgressive potential

## Abstract

Northeast China is a major soybean production region in China. A representative sample of the Northeast China soybean germplasm population (NECSGP) composed of 361 accessions was evaluated for their seed protein content (SPC) in Tieling, Northeast China. This SPC varied greatly, with a mean SPC of 40.77%, ranging from 36.60 to 46.07%, but it was lower than that of the Chinese soybean landrace population (43.10%, ranging from 37.51 to 50.46%). The SPC increased slightly from 40.32–40.97% in the old maturity groups (MG, MGIII + II + I) to 40.93–41.58% in the new MGs (MG0 + 00 + 000). The restricted two-stage multi-locus genome-wide association study (RTM-GWAS) with 15,501 SNP linkage-disequilibrium block (SNPLDB) markers identified 73 SPC quantitative trait loci (QTLs) with 273 alleles, explaining 71.70% of the phenotypic variation, wherein 28 QTLs were new ones. The evolutionary changes of QTL-allele structures from old MGs to new MGs were analyzed, and 97.79% of the alleles in new MGs were inherited from the old MGs and 2.21% were new. The small amount of new positive allele emergence and possible recombination between alleles might explain the slight SPC increase in the new MGs. The prediction of recombination potentials in the SPC of all the possible crosses indicated that the mean of SPC overall crosses was 43.29% (+2.52%) and the maximum was 50.00% (+9.23%) in the SPC, and the maximum transgressive potential was 3.93%, suggesting that SPC breeding potentials do exist in the NECSGP. A total of 120 candidate genes were annotated and functionally classified into 13 categories, indicating that SPC is a complex trait conferred by a gene network.

## Introduction

Soybean [*Glycine max* (L.) Merr.], which originated in ancient central China, is a traditional crop rich in seed protein (SPC, ~40%) and oil content (~20%) (Zhang et al., 2015a). It had been disseminated to Liao-river valleys in Northeast China (NEC) more than 2000 years ago and has expanded to the whole NEC in recent centuries. NEC is currently the major production area for soybean and a major source of soybean commodities for soy food processing, including tofu products and protein isolates for human food and animal feed in China (Warrington et al., 2015). However, the SPC of commercial soybeans in NEC is about 39 to 42%, less than in central and southern China (about 40 to 45%). The food processing companies demand increased SPC in commercial soybean production, especially in NEC. To improve soybean SPC, the first step is to investigate the phenotypic and genetic variation of the soybean germplasm to estimate whether there is genetic potential available to be utilized. Liu et al. (2020) found that the NEC soybean germplasm population (NECSGP) was derived from the original population from central China, with several newly derived and introduced accessions added during the recent century. The NECSGP was genetically clustered together with those from the north and south Americas and was the major germplasm source of the soybeans in the Americas, where ~85% of world soybeans are produced at present (Fu et al., 2020a). Thus, exploring the genetic basis of the SPC in NEC soybean germplasm is of great significance not only for NEC soybean production but also for global soybean production.

SPC is a quantitative trait controlled by many genes and is also affected by the environment (Hwang et al., 2014). There were 248 SPC QTLs (quantitative trait loci) reported at SoyBase (https://soybase.org). These SPC QTLs were detected by using linkage mapping procedures (Zhang et al., 2015a) and are mainly located on chromosomes 4, 5, 7, 8, 14, 15, 18, 19, and 20. Karikari et al. (2019) identified 25 SPC QTLs in a linkage mapping study under a single-locus model using a recombinant inbred line (RIL) population derived from *Linhefenqingdou* and *Meng8206*, in which *qPro-7-1* was detected simultaneously in three environments, with an average phenotypic variance (PV) of 19.01%. Among these QTLs, 10 QTLs were newly detected and the PV of 12 QTLs were all greater than 10%, with the lowest PV of 8.97%. Teng et al. (2017) identified 8 SPC QTLs in 12 environments using the RIL population derived from *Dongnong46*×*L-100*, in which *qPR-2, qPR-3, qPR-5, qPR-7*, and *qPR-8* were detected simultaneously in 6, 8, 7, 6, 7 environments, respectively. The candidate gene *Glyma.20g085100* underlying the major SPC QTL on chromosome 20 was mapped and cloned (Fliege et al., 2022). The haplotype variation at this major QTL in wild and domesticated soybean was also explored using a germplasm population consisting of 985 accessions (Marsh et al., 2022).

QTL detection based on linkage mapping usually involves only two parental lines, such as the RIL population, where the genetic variation and mapping resolution are quite limited. Association mapping based on natural germplasm populations provides a powerful method for genome-wide QTL detection. By using association mapping in a large germplasm population consisting of 12,116 cultivated soybean accessions, Bandillo et al. (2015) detected 19 SNPs associated with SPC mainly on chromosome 15 (3.82 – 3.96 Mb) and chromosome 20 (29.59 – 31.97 Mb). Sonah et al. (2015) reported that eight regions were significantly associated with SPC based on 139 soybean accessions. The region on chromosome 8 between 45.5 and 46.9 Mb had the largest number of significantly associated SNPs, while there was only one associated SNP on chromosome 19 (50.4 Mb) and chromosome 20 (10.0 Mb). Zhang et al. (2017) reported that 15 loci were associated with SPC, with their phenotypic contribution ranging from 17.4 to 29.2%, and the candidate gene *Glyma.13g123500* was highly expressed during seed development.

However, the previous association mapping studies were mainly based on single-locus model analysis. Each genome-wide marker was tested independently for its association with a quantitative trait. The Bonferroni-adjusted threshold was applied to correct the multiple testing problem (Sul et al., 2018; Tam et al., 2019). The stringent threshold in the single-locus model largely reduces the false positives and leads to many false negatives (Benjamini and Yekutieli, 2001). Furthermore, the bi-allelic SNP makers are usually used in association mapping. Therefore, the multiple alleles of a QTL that widely existed in germplasm populations cannot be detected directly (Nachman, 2001; Yang et al., 2012). He et al. (2017) proposed the restricted two-stage multi-locus model genome-wide association analysis (RTM-GWAS) method to thoroughly detect QTLs and their multiple alleles. This procedure has the following merits: (i) Use the SNP linkage disequilibrium blocks (SNPLDB) as markers with multiple haplotypes to fulfill the multiple allele characteristic in natural populations. (ii) Use two-stage GWAS for efficient association analysis, that is, first stage GWAS under single locus model for preselecting markers and second stage multi-locus model stepwise regression for identifying QTLs-alleles with trait heritability (*h*^2^) as the upper limit of QTL total contribution to reduce false positives and negatives. (iii) Use normal *p*-value without excessive Bonferroni correction. All the detected QTLs are tested jointly under the multi-locus model. (iv) Use plot-based phenotype data to minimize the error amount through experiment design to raise the QTL-identification precision (He and Gai, 2020; Liu et al., 2021). Therefore, RTM-GWAS can provide a high QTL detection power and efficiency. The QTL-allele matrix is further established based on the results as a compact form of the population's genetic structure and individual accessions. This procedure has been demonstrated for its effectiveness in a series of soybean germplasm studies and even bi-parental population studies, such as on 100-seed weight (Zhang et al., 2015b), seed isoflavone content (Meng et al., 2016), days to flowering (Liu et al., 2021), and main stem node number (Fahim et al., 2021). Using RTM-GWAS, 26 SPC QTLs were detected based on 279 soybean accessions from China's Yangtze and Huaihe River Valley (Li et al., 2019). These QTLs accounted for 58.3% of the phenotypic variation, with *qProt-20-3* having the highest PV (16%). Li et al. (2020) detected 90 SPC QTLs using RTM-GWAS in a soybean nested association mapping population. Twenty QTLs were newly detected, and *Glyma20g24830* and *Glyma18g03540* were annotated as important candidate genes for SPC.

The germplasm collection of an ecoregion is historically accumulated and may vary from time to time due to additions and losses. The germplasm accessions used for genetic studies should represent the ecoregion population so that the conclusions drawn can explain the real population rather than some unknown population. In the present study, we recollected soybean accessions from all the research institutions in NEC and then chose those from all subregions and historical reserves to form a representative soybean germplasm sample in NEC. In addition, NEC covers a wide range of latitudes. For evaluation of SPC under the same environment, the experiment site should be at a place where all kinds of the maturity group soybeans can mature naturally. Based on the above considerations, this study aimed at (i) exploring the SPC variation in the NECSGP, (ii) exploring the SPC QTL-allele system in the NECSGP, (iii) characterizing the genetic mechanism in the evolutionary process from late to early maturity groups (MGs) in NEC, (iv) exploring the QTL-allele recombination potential for optimal cross design in NEC, and (v) inferring the SPC candidate gene system.

## Materials and Methods

### Plant Materials and Field Experiments

A total of 361 representative soybean accessions were collected and chosen from the NECSGP. The accessions covered six MGs, including MG III, MG II, MG I, MG 0, MG 00, and MG000 (Fu et al., 2020a). ln 2013–2014, these accessions were tested at Tieling, Northeast China. The “Blocks in Replication” design was used, with 4 hills in a row-plot, 1.0 m in length, and 1.0 m row space. According to their MGs, the accessions were grouped into six blocks and four replications were implemented each year. At the maturity (R8) stage, the plants in each plot were threshed and dried after harvest, and then the SPC was measured by using the FOSS NearInfared grain analyzer Infratec 1241.

### Statistical Analysis

The experimental data were analyzed using a joint randomized block design analysis as an approximation for simplicity. The analysis of variance was performed using the PROC GLM procedure of the SAS/STAT software (SAS Institute Inc., Cary, NC, USA). The linear model was


$$\begin{array}{l} y_{ijk}=\mu +t_{i}+r_{j\left(i\right)}+g_{k}+{\left(gt\right)}_{ik}+\varepsilon_{ijk}, \end{array}$$

where *y*_*ijk*_ is the phenotype value of the *k*-th accession for the *j*-th replication in the *i*-th environment, μ is the population mean, *t*_*i*_ is the effect of the *i*-th environment, *r*__*j*_(i)_ is the effect of the *j*-th replication in the *i*-th environment, *g*_*k*_ is the effect of the *k*-th accession, (*gt*)_*ik*_ is the interaction effect between accession and environment, and ε_*ijk*_ is the random error following *N*(0, σ^2^). Except that the effect of accession was considered fixed, all other effects were considered random. The trait heritability for the single environment and multiple environments was estimated, respectively, as


$$\begin{array}{lll} h^{2} & = & \sigma_{g}^{2}/\left(\sigma_{g}^{2}+\sigma^{2}/n_{r}\right)\text{and}\\ h^{2} & = & \sigma_{g}^{2}/\left[\sigma_{g}^{2}+\sigma_{gt}^{2}/n_{t}+\sigma^{2}/\left(n_{t}\times n_{r}\right)\right] \end{array}$$

where $\sigma_{g}^{2}$ is the genotype variance, $\sigma_{gt}^{2}$ is the genotype and year interaction variance, σ^2^ is the error variance, *n*_*t*_ is the number of years, and *n*_*r*_ is the number of replications. The variance components were estimated using the PROC MIXED procedure of the SAS/STAT software (SAS Institute Inc., Cary, NC, USA). The genetic coefficient of variation (*GCV*) was calculated as *GCV* = σ_g_/μ.

### SNP Genotyping, SNPLDB Assembly, and RTM-GWAS Analysis

The genotype data of the 361 accessions were obtained from Fu et al. (2020a), and the accessions were sequenced with restriction site-associated DNA sequencing technology (RAD-seq) (Miller et al., 2007) at BGI tech, Shenzhen, China. All sequence reads were aligned against the reference genome Wm82.a1.v1.1 (Schmutz et al., 2010) using the SOAP2 (Li et al., 2009) software. The RealSFS (Yi et al., 2010) was used for SNP calling. The SNPs with missing rat e >20%, heterozygosity rate >20%, and minor allele frequency (MAF) < 0.01 were filtered out. The missing genotypes were then imputed using the fastPHASE software (Scheet and Stephens, 2006). Finally, 82,966 high-quality SNPs were obtained. The SNPs were then grouped into SNPLDB markers based on genomic block partition using the RTM-GWAS software, with haplotypes as their alleles and an LD threshold of *D*'>0.7 (He et al., 2017). A total of 15,501 SNPLDBs were identified in the NECSGP.

The RTM-GWAS procedure was used to dissect the genetic constitution underlying the SPC variation in the NECSGP, in which the genetic similarity coefficients (GSC) between accessions were calculated based on genome-wide SNPLDBs. The top 10 eigenvectors of the GSC matrix were used as the covariates to correct the population structure bias. A threshold of 0.05 was used at the first stage of RTM-GWAS for candidate marker preselection, and the significance level was set to 0.01 for stepwise regression at the second stage of RTM-GWAS. The detected QTLs (associated SNPLDBs) with their allele effects for each accession were used to establish an SPC QTL-allele matrix of the NECSGP for further analysis (He et al., 2017). Compared to the QTLs reported in SoyBase (https://soybase.org), a QTL was considered overlapped if its physical position was located in the same region as that in the SoyBase.

### Transgressive Potential Prediction and Optimal Cross Design in the NECSGP

Based on the SPC QTL-allele matrix, all possible 64,980 single crosses (361 × 360/2) were generated *in silico* (He et al., 2017). Both linkage and independent models were used to analyze the recombination potential of SPC in the NECSGP. In the linkage model, the number of crossovers on each chromosome was simulated randomly according to the Poisson distribution with chromosome length as a parameter, while in the independent model, all genetic loci were considered independent of each other. The predicted genotypic SPC value was calculated for each cross based on 2,000 homozygous progenies derived from F_2_ individuals through continuous selfing. The 95th percentile value was used as the predicted value for the recombination potential of each cross. The *cross* program (https://gitee.com/njau-sri/cross) was used for simulation. Based on the recombination potential analysis of individual crosses, the transgressive potential was predicted for crosses within an MG and crosses between MGs. The highest SPC of accessions observed in the MG(s) was used as a check to indicate the transgressive potential of a cross.

### Candidate Gene Prediction

The steps of candidate gene prediction were as follows: (1) the genomic interval of a detected QTL (SNPLDB) was extended by 200 kb at both ends according to the LD decay distance in cultivated soybean populations; (2) within the genomic interval, the genes of the reference genome Wm82.a1.v1.1 were retrieved from SoyBase (https://soybase.org); and (3) the independence between a QTL and gene(s) within the QTL interval was tested using Chi-square criterion at a significance level of 0.05. The Gene Ontology (GO) annotations of genes were retrieved from SoyBase.

## Results

### Features of SPC Variation in the NECSGP

The joint analysis of variance (ANOVA) over two environments indicated significant SPC variation among the genotypes (accessions) and the genotype-by-environment interactions (Supplementary Table 1). The SPC of the NECSGP in Tieling ranged from 36.60 to 46.07%, with an average SPC of 40.77%. The heritability of SPC over two environments was estimated as 83.05%, with the *GCV* of 3.43% and the genotype-by-environment interaction (GEI) heritability of 11.56%, indicating the phenotypic SPC variation in the NECSGP was mainly caused by genotypic variation and affected slightly by GEI (Table 1). The SPC in NECSGP varied greatly but was not as wide as that in the Chinese soybean landrace population, where the SPC variation range was 37.51 to 50.46%, with an average of 43.10% (Zhang et al., 2018).

**Table 1:** Frequency distribution and descriptive statistics for SPC in the NECSGP.

**Maturity group**	**Mid-point of SPC (%)**										***N***	**Mean (%)**	**Range (%)**	***h*^2^ (%)**	
	**37**	**38**	**39**	**40**	**41**	**42**	**43**	**44**	**45**	**46**				**G**	**G × E**
III			3	5	6	5	1	1			21	40.97 b	38.54–43.93		
II	1	4	8	8	14	2	6				43	40.33 c	37.01–43.48		
I	1	2	21	19	25	7	4				79	40.32 c	36.60–43.16		
0		4	12	45	51	25	15	4	1		157	40.93 b	37.56–44.71		
00			4	14	10	11	5	1			45	41.01 b	38.88–44.00		
000			1	3	4	6	0	1	0	1	16	41.58 a	39.19–46.07		
Entire	2	10	49	94	110	56	31	7	1	1	361	40.77	36.60–46.07	83.05	11.56

*NECSGP, Northeast China soybean germplasm population; SPC, seed protein content; N, number of materials; a, b, and c denote Duncan's new multiple range test, the means with different letters indicating significance at P < 0.05; G, genotype; G × E, genotype by environment interaction*.

The results also showed that the difference in SPC among MGs was significant but relatively not large. The average SPC ranged from 40.32 to 41.58% among different MGs (Table 1). There was a slight increase in average SPC from late MGs (III + II + I) to early MGs (000 + 00 + 0) or from longer growth period to shorter growth period. MG II and I exhibited the lowest SPC while MG 000 exhibited the highest SPC. This trend implied that the SPC might retain at least a similar level of the NECSGP in breeding earlier maturing soybean varieties further northward. In this case, figuring out whether there is further SPC improvement potential depends on exploring the genetic recombination potential based on a relatively thorough exploration of the QTL-allele/gene-allele constitution of the NECSGP.

### Identification of the SPC QTL-Allele System in the NECSGP

**Figure 1:**
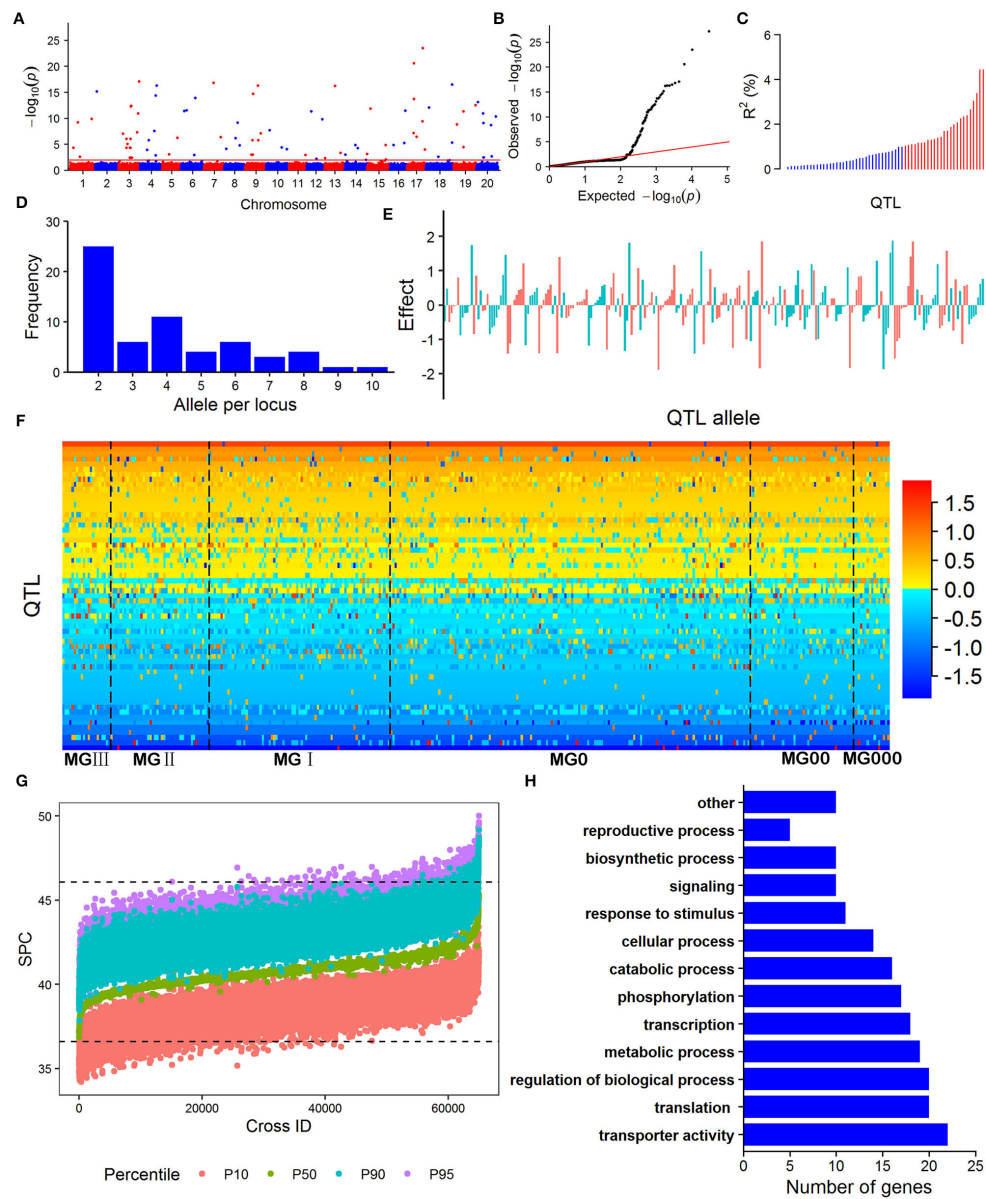
The SPC QTL-allele information of the Northeast China soybean germplasm population obtained from RTM-GWAS. **(A)** Manhattan plot; **(B)** Quantile - quantile plot; **(C)** The phenotypic contribution of the 61 main-effect QTLs, blue bars denote small-contribution QTL (*R*^2^ < 1%), red bars represent large-contribution QTL (*R*^2^ ≥ 1%); **(D)** The frequency distribution of allele number per locus for the 61 main-effect QTLs; **(E)** SPC allele effects of the 61 main-effect QTLs; **(F)** QTL-matrix of SPC in the NECSGP; **(G)** Predicted SPC of progenies in possible crosses; **(H)** Gene Ontology (GO) biological process annotations of the candidate genes for SPC QTLs in the NECSGP. “other” GO biological processes include snoRNA localization, localization, anatomical structure development, post-embryonic development, multicellular organism development, activation of protein kinase activity, Golgi organization, chloroplast organization, macromolecule methylation, and methylation.

The RTM-GWAS with QTL-by-environment interaction (QEI) model was used to identify the SPC QTL-allele constitution since GEI was significant in ANOVA. A total of 15,501 SNPLDBs were constructed based on 82,966 SNPs. There were 8,780 SNPLDBs containing only a single SNP (S.SNPLDB) and 6,721 SNPLDBs containing multiple SNPs (M.SNPLDB). The number of alleles for M.SNPLDB ranged from 2 to 10 with an average of 3.5, while 1,792 M.SNPLDBs had only two alleles. At the first stage of RTM-GWAS under the single-locus model, 9,078 SNPLDBs were preselected from a total of 15,501 SNPLDBs. At the second stage in stepwise regression under the multiple-locus model, out of the preselected SNPLDBs, a total of 73 with 273 haplotypes/alleles passed the model test and were detected to be associated with SPC (Figures 1A,B). Among the 73 QTLs, 36 QTLs had the main effect only, 12 QTLs had only QEI effect, and 25 QTLs had both the main and QEI effect (Table 2). The 73 QTLs accounted for 71.70% of the phenotypic variation (PV). The 61 main effect QTLs with 240 alleles explained 62.72% PV and the 37 QEI QTLs with 138 alleles explained 8.98% PV. As indicated in Figure 1C, the phenotypic contribution of the main effect of QTLs varied continuously. When 1% PV was used as an artificial threshold for QTL classification, 61 QTLs could be classified as 25 large contribution QTLs (LC, *R*^2^ ≥ 1%) with 105 alleles and 36 small contribution QTLs (SC, *R*^2^ < 1%) with 135 alleles (Table 2). In the same way, all the QEI QTLs were classified into 37 SCs with 138 alleles, and there were no LCs.

**Table 2:** QTLs/SNPLDBs associated with SPC in the NECSGP.

**QTL**	**AN**	**Model**	**QTL**		**QTL × Env**.		**QTL**	**AN**	**Model**	**QTL**		**QTL × Env**.	
		**–lg*P***	**–lg*P***	***R*^2^(%)**	**–lg*P***	***R*^2^ (%)**			**–lg*P***	**–lg*P***	***R*^2^(%)**	**–lg*P***	***R*^2^ (%)**
*q-Prot-1-1*	2	4.29			5.33	0.22	*q-Prot-11-1*	2	2.89	2.89	0.11		
*q-Prot-1-2*	2	9.18	13.24	0.61	3.31	0.13	*q-Prot-12-1*	4	11.33	20.84	1.09	2.00	0.12
*q-Prot-1-3*	2	2.60			3.24	0.13	*q-Prot-12-2*	2	2.17			2.35	0.09
*q-Prot-1-4*	5	9.82	17.81	0.97			*q-Prot-12-3*	2	9.77	16.63	0.78		
*q-Prot-2-1*	6	15.17	41.01	2.24			*q-Prot-13-1*	2	16.17	40.01	1.97	2.81	0.11
*q-Prot-3-1*	6	7.02	7.50	0.46	3.76	0.26	*q-Prot-13-2*	5	3.32			2.77	0.18
*q-Prot-3-2*	3	5.05	2.07	0.10	5.33	0.26	*q-Prot-14-1*	3	2.94	3.98	0.20		
*q-Prot-3-3*	8	12.22	23.15	1.36	9.18	0.61	*q-Prot-14-2*	2	4.82	2.23	0.08	4.53	0.19
*q-Prot-3-4*	2	12.33	26.81	1.29			*q-Prot-14-3*	2	4.25			4.26	0.17
*q-Prot-3-5*	8	7.24	11.56	0.74			*q-Prot-15-1*	2	3.08	3.93	0.16		
*q-Prot-3-6*	2	10.88	21.46	1.02			*q-Prot-15-2*	6	11.82	23.17	1.29	5.46	0.35
*q-Prot-3-7*	6	17.08	55.56	3.03			*q-Prot-15-3*	3	4.82	6.58	0.32		
*q-Prot-4-1*	10	3.87	5.15	0.43			*q-Prot-15-4*	2	2.04			2.47	0.09
*q-Prot-4-2*	2	5.78	4.14	0.17	4.34	0.18	*q-Prot-16-1*	4	4.83			7.14	0.39
*q-Prot-4-3*	8	7.54	10.42	0.68	2.30	0.22	*q-Prot-16-2*	2	5.19	5.63	0.24		
*q-Prot-4-4*	2	14.34	34.21	1.67			*q-Prot-16-3*	4	11.42	22.41	1.17		
*q-Prot-4-5*	2	2.83	3.14	0.12			*q-Prot-17-1*	4	7.13	8.56	0.46	2.39	0.14
*q-Prot-4-6*	4	16.27	44.42	2.32			*q-Prot-17-2*	4	20.59	63.37	3.36		
*q-Prot-5-1*	2	3.04			3.87	0.16	*q-Prot-17-3*	6	6.38	9.96	0.60		
*q-Prot-5-2*	6	6.20			7.91	0.49	*q-Prot-17-4*	2	9.41	15.50	0.72		
*q-Prot-6-1*	4	11.37	20.11	1.05	5.41	0.30	*q-Prot-17-5*	6	23.46	80.02	4.42		
*q-Prot-6-2*	2	11.48	24.00	1.15			*q-Prot-17-6*	7	3.94	3.72	0.28	2.02	0.18
*q-Prot-6-3*	2	2.91	3.50	0.14			*q-Prot-18-1*	9	12.02	23.54	1.42		
*q-Prot-6-4*	3	13.90	33.44	1.69			*q-Prot-18-2*	2	2.60	2.70	0.10		
*q-Prot-7-1*	2	16.82	41.48	2.05			*q-Prot-18-3*	5	16.45	44.25	2.37	6.33	0.37
*q-Prot-7-2*	4	6.35	5.06	0.28	4.49	0.25	*q-Prot-19-1*	2	8.76	4.61	0.19	9.69	0.43
*q-Prot-8-1*	3	4.11	4.43	0.22			*q-Prot-19-2*	7	11.33	23.02	1.32	2.36	0.20
*q-Prot-8-2*	2	6.13	3.69	0.15	6.14	0.26	*q-Prot-19-3*	2	4.40	4.96	0.21		
*q-Prot-8-3*	8	9.12	15.77	0.97			*q-Prot-19-4*	4	27.19	82.27	4.43	8.09	0.43
*q-Prot-8-4*	2	4.74			6.33	0.27	*q-Prot-20-1*	2	13.07	24.11	1.15	5.75	0.24
*q-Prot-9-1*	5	5.72	6.14	0.36	2.40	0.16	*q-Prot-20-2*	3	10.94	21.74	1.09		
*q-Prot-9-2*	4	14.66	35.57	1.85			*q-Prot-20-3*	7	9.05	11.27	0.69	6.99	0.46
*q-Prot-9-3*	4	16.28	50.34	2.64			*q-Prot-20-4*	2	8.65	11.40	0.52	3.82	0.15
*q-Prot-9-4*	2	5.73	7.69	0.34			*q-Prot-20-5*	2	2.61			2.48	0.09
*q-Prot-9-5*	5	7.05	4.52	0.28	7.40	0.43	*q-Prot-20-6*	2	10.29	18.00	0.84		
*q-Prot-10-1*	4	7.66	12.27	0.65			LC-QTL	105		25	48.42		
*q-Prot-10-2*	2	4.35	3.78	0.15	2.48	0.09	SC-QTL	135		36	14.30	37	8.98
*q-Prot-10-3*	2	4.03			4.38	0.18	Total	273	73	61	62.72	37	8.98

*NECSGP, Northeast China soybean germplasm population; SPC, seed protein content; QTL, the name of associated SNPLDB, e.g., for q-Prot-10-1, “q” means QTL, “Prot” means protein, 10 is the chromosome number, and 1 is the position order; AN, number of alleles*.

The main effect QTLs are located on all chromosomes except Chr. 5, seven main effect QTLs on Chr. 3, six on Chr. 4 and 17, and one on Chr. 2, 11, and 13, respectively. The number of alleles for each main effect QTL ranged from 2 to 10 (Figure 1D) with allele effects ranging from −1.89 to 1.88 (Figure 1E; Supplementary Table 2). Compared to the previously reported SPC QTLs, 45 out of the 73 detected QTLs overlapped with those reported in the SoyBase (http://soybase.org), including the two QTL hotspots on Chr. 9 and 20. The remaining 28 QTLs were newly found in the present study (Supplementary Table 3). The 61 SPC main effect QTLs and their allele effects for each of the 361 accessions were organized as a QTL-allele matrix (Figure 1F), a compact form of the genetic constitution of the NECSGP. At the same time, the QTL-allele matrix can be further separated into submatrices corresponding to the six MGs (Figure 1F). The QEI QTL-allele data set can also be organized into a matrix if it is needed. But the environmental factor in the present study varied randomly, and no fixed environmental parameter was available to provide useful information in breeding for SPC improvement. Therefore, the QEI information was not used in further analysis.

### SPC QTL-Allele Changes in the Evolution From Late to Early MGs in NECSGP

The above results indicated that the SPC in NECSGP slightly increased with the development of earlier soybean MGs due to the further northward dissemination after its introduction into the Liao-River valleys. During this artificial evolutionary process, the QTLs-alleles also changed. Some original alleles were passed down, some new ones emerged, and some old ones were excluded. New recombinants were formed, as indicated in Figure 1F. To analyze the QTL-allele changes from MG III + II + I to earlier MGs, the dynamic QTL-allele data were listed in the upper part of Table 3 and the summary statistics in the lower part. All the detected main effect and QEI effect data of the 73 loci with their 273 alleles were included since all are involved in the evolutionary process.

**Table 3:** The SPC QTL-allele changes among maturity groups.

**QTL**	**a1**	**a2**	**a3**	**a4**	**a5**	**a6**	**a7**	**a8**	**a9**	**a10**	**QTL**	**a1**	**a2**	**a3**	**a4**	**a5**	**a6**	**a7**	**a8**	**a9**	**a10**
***q-Prot-1-1***	z										***q-Prot-10-3***		y								
***q-Prot-1-2***		yz									***q-Prot-11-1***										
***q-Prot-1-3***	XY										***q-Prot-12-1***										
***q-Prot-1-4***											***q-Prot-12-2***		y								
***q-Prot-2-1***	y		z		yz	y					***q-Prot-12-3***		z								
***q-Prot-3-1***		y	y								***q-Prot-13-1***										
***q-Prot-3-2***	z	yz									***q-Prot-13-2***					yz					
***q-Prot-3-3***						yz	z	yz			***q-Prot-14-1***	yz	z								
***q-Prot-3-4***	z										***q-Prot-14-2***	yz									
***q-Prot-3-5***	yz	yz	y		z			z			***q-Prot-14-3***										
***q-Prot-3-6***											***q-Prot-15-1***										
***q-Prot-3-7***	yz		z	z							***q-Prot-15-2***		z	z			yz				
***q-Prot-4-1***		yz	yz	z	z	xyz	y			z	***q-Prot-15-3***			z							
***q-Prot-4-2***	z										***q-Prot-15-4***	z									
***q-Prot-4-3***	yz	XY		z			y	z			***q-Prot-16-1***										
***q-Prot-4-4***	y										***q-Prot-16-2***		XY								
***q-Prot-4-5***		z									***q-Prot-16-3***				z						
***q-Prot-4-6***			yz								***q-Prot-17-1***										
***q-Prot-5-1***	z										***q-Prot-17-2***		y								
***q-Prot-5-2***	z				yz	yz					***q-Prot-17-3***	z	z	y							
***q-Prot-6-1***				XYZ							***q-Prot-17-4***		z								
***q-Prot-6-2***											***q-Prot-17-5***	yz	yz		z						
***q-Prot-6-3***	z										***q-Prot-17-6***			z				yz			
***q-Prot-6-4***			y								***q-Prot-18-1***			y		z			z	z	
***q-Prot-7-1***		y									***q-Prot-18-2***		xyz								
***q-Prot-7-2***	z										***q-Prot-18-3***		yz		z						
***q-Prot-8-1***	z										***q-Prot-19-1***	yz									
***q-Prot-8-2***		X									***q-Prot-19-2***						y	yz			
***q-Prot-8-3***	yz	z		yz			yz	z			***q-Prot-19-3***										
***q-Prot-8-4***		y									***q-Prot-19-4***	z	z		yz						
***q-Prot-9-1***	z	z									***q-Prot-20-1***		XY								
***q-Prot-9-2***	xz			z							***q-Prot-20-2***	yz									
***q-Prot-9-3***	z		z	z							***q-Prot-20-3***	z			z		z	y			
***q-Prot-9-4***		xz									***q-Prot-20-4***	z									
***q-Prot-9-5***		z									***q-Prot-20-5***										
***q-Prot-10-1***	yz	xz									***q-Prot-20-6***										
***q-Prot-10-2***		H																			
**Maturity group**			**Total allele**				**Inherent allele**				**Emerged allele**				**Excluded allele**	
			**Allele**	**QTL**			**Allele**	**QTL**			**Allele**	**QTL**			**Allele**	**QTL**
I + II + III			267 (142,125)	73							
I + II + III vs. 0			268 (143,125)	73			262 (141,121)	73			6 (2,4)	6			5 (1,4)	5
I + II + III vs. 00			222 (120,102)	73			217 (118,99)	73			5 (2,3)	5			50 (24,26)	35
I + II + III vs. 000			180 (95,85)	73			179 (95,84)	73			1 (0,1)	1			88(47,41)	46
I + II + III vs. 0 + 00 + 000			271 (144,127)	73			265 (142,123)	73			6 (2,4)	6			2 (0,2)	2

*In the upper part: a1–a10 are the alleles of a QTL; the white cells represent negative effect alleles and the gray ones represent positive effect alleles; “x,” “y,” “z,” respectively, represent alleles excluded in MG0, MG00, MG000 (vs. MGI + II + III); “X,” “Y,” “Z,” respectively, represent alleles emerged in MG0, MG00, MG000 (but not existing in MGI + II + III)*.

*In the lower part: the number outside parentheses is the total of alleles; the numbers in parentheses are the number of negative effect alleles and positive effect alleles, respectively; Inherent allele means alleles passed from the compared MG; Emerged allele means the alleles new to the partner of I + II + III, 0, and 00, respectively; Excluded allele means the alleles excluded in the compared MG*.

In comparison to the old MGs (MG I~III), only one allele of *q*-*Prot*-*6*-*1* (a4) emerged in all the three new MGs (MG 0~000) and only two alleles of *q*-*Prot*-*4*-*1* (a6) and *q*-*Prot*-*18*-*2* (a2) were excluded in all the three new MGs (Table 3 upper part). There were different patterns of allele changes during the artificial evolutionary process from the old MGs to each of the new MGs. From the old MGs to the new MG 0, five alleles (one negative and four positives) of five QTLs were excluded and six alleles (two negatives and four positives) of six QTLs emerged. The number of emerged alleles was much less than that of excluded alleles from the old MGs to the new MG 00 and 000, that is, 50 alleles (24 negative and 26 positive) of 35 QTLs were excluded and five alleles (two negatives and three positives) of five QTLs emerged from the old MGs to the new MG 00, and 88 alleles (47 negatives and 41 positives) of 46 QTLs were excluded and one allele (positive effect) emerged from the old MGs to MG 000. With the shortening of the growth period, the number of excluded alleles increased and the number of emerged new alleles decreased.

Due to limited sample sizes, there might be some fluctuation in new MGs. Thus, only the comparison was made between the old MGs (III + II + I) and emerging MGs (0 + 00 + 000). There were 267 (142 negatives and 125 positives) alleles in the old MGs, of which 265 (142 negatives and 123 positives) alleles were inherited in the new MGs. Or in other words, 97.79% (265/271) alleles in new MGs were inherited from the old MGs, while six (2.21%) alleles (two negatives and four positives) emerged and two (2/267=0.75%) alleles of positive effect were excluded. Thus, the most alleles of the SPC QTLs in the old MGs were reserved in the new MGs, with only eight alleles changed. These changes in alleles caused an increase in SPC from 40.32–40.97% in the old MGs to 40.93–41.58% in the new MGs. The four alleles of the positive effect that emerged were responsible for the SPC increase as no alleles of negative effect were excluded. Accordingly, the evolutionary motivation of the slight increase in SPC of the new MGs compared to the old MGs might be due to the emergence of new alleles and possible recombination between inherited alleles rather than the exclusion of alleles. Thus, the following text will focus on the recombination or transgressive potential of the NECSGP.

### Prediction of Allele Recombination Potential for Optimal Cross Design in the NECSGP

The genotypes of 2,000 homozygous progenies were simulated for each of the 64,980 possible crosses among the 361 soybean accessions in the NECSGP, then the SPC of the progenies was predicted based on the SPC QTL-allele matrix in the population. In this study, as the linkage and independent model results were very similar, only the simulation results of the linkage model were used to explore the allele recombination potential. For each cross, the SPC percentile of the progeny population was used as an indicator of recombination potential between alleles. As shown in Figure 1G, transgressive recombination for SPC existed in the NECSGP. Using the 95th percentile, the predicted SPC of the 64,980 crosses ranged from 37.84 to 50.00%, with an average of 43.29%, and 1,803 crosses showed higher SPC than the maximum SPC (46.07%) in the NECSGP (Table 4). Transgressive recombination for SPC was observed both for crosses within and between MG(s). Using the 95th percentile, 534 crosses within MGs and 1,269 crosses between MGs showed higher SPC than the maximum SPC in the NECSGP. The average SPC of predicted crosses within and between maturity groups were similar, but the maximum SPC between MGs was higher than that within MGs (Table 4).

**Table 4:** The predicted SPC of simple crosses within and between maturity groups.

**Maturity group (MG)**	**Maximum SPC**	**Total no. of crosses**	**Predicted SPC^a^**			**Superior crosses^b^**
			**Mean**	**Min**.	**Max**.	
I + II + III	43.93	10,153	42.97	38.85	48.37	171
0	44.71	12,246	43.38	37.84	48.91	318
00	44.00	990	43.43	40.36	48.01	22
000	46.07	120	44.62	41.43	48.72	23
I + II + III vs. 0	44.71	22,451	43.20	38.69	48.71	483
I + II + III vs. 00	44.00	6,435	43.23	39.41	48.81	123
I + II + III vs. 000	46.07	2,288	43.85	39.35	49.63	184
0 vs. 00	44.71	7,065	43.41	40.38	48.51	177
0 vs. 000	46.07	2,512	44.03	40.33	50.00	235
00 vs. 000	46.07	720	44.02	40.39	49.48	67
Within^c^	46.07	23,509	43.21	37.84	48.91	534
Between^d^	46.07	41,471	43.34	38.69	50.00	1,269
Entire^e^	46.07	64,980	43.29	37.84	50.00	1,803

a *Predicted SPC is the 95^th^ percentile of predicted SPC in a simple cross*.

b *Superior crosses mean the number of crosses with their 95^th^ percentile value more than the maximum SPC of the MG*.

c *“Within” means within all MGs*.

d *“Between” means MG I + II + III and MG 0 + 00 + 000*.

e *“Entire” means all possible simple crosses in the whole population*.

For crosses within MGs, 171, 318, 22, and 23 crosses within MG I + II + III, 0, 00, and 000, respectively, showed higher SPC than the maximum SPC in the NECSGP. The predicted SPC for each group was similar, with the maximum SPC ranging from 48.01 to 48.91%. For crosses between MGs, the predicted SPC varied, with the maximum SPC ranging from 48.51 to 50.00%. The crosses between MG 0 and 000 exhibited the maximum recombination potential, and the SPC of crosses between new MGs was slightly higher than that between old MGs and new MGs (Table 4).

The above results indicated allele recombination potential for SPC improvement in terms of the 95th percentile at the NECSGP level. The average recombination potential for SPC improvement was estimated as 2.52% (=43.29–40.77), with the maximum recombination potential as 9.23% (=50.00–40.77) and the maximum transgressive potential as 3.93% (=50.00–46.07). From the individual MG level, the above three comparisons varied similarly. For example, in MG 0, the mean recombination potential was estimated as 2.45% (=43.38–40.93) in MG 0, with the maximum recombination potential as 7.98% (=48.91–40.93) and the maximum transgressive potential as 4.20% (=48.91–44.71). Thus, there were superior recombination and transgressive potential within/among the MGs in the NECSGP. The potential for SPC improvement exists in the population and remains to be explored according to the SPC QTL-allele constitution of the NECSGP.

The five best crosses were selected for each MG and the entire NECSGP (Table 5). The cross between L54 (MG 000) and L5 (MG 0) exhibited the highest 95th percentile of the predicted SPC (50.00%), with an 8.53% increase in SPC compared with the maximum SPC in the NECSGP. Although the recombination potential was relatively limited within MG, it may also reach up to 50% under intensive selection, as indicated by the 99th percentile. For example, the 99th percentile of predicted SPC of the cross L329 × L5 was 50.04%, and that of L54 × L5 was as high as 51.54%. Thus, according to the QTL-allele matrix, the optimal or best crosses can be designed readily.

**Table 5:** Optimal crosses for high SPC in different maturity groups (%).

**Maturity group**	**P1**	**P2**	**Y1**	**Y2**	**Mean**	**SD**	**P90**	**P95**	**P99**
I + II + III	L177	L326	43.16	42.34	42.77	3.30	47.06	48.13	50.11
	L115	L326	43.48	42.34	42.89	3.07	46.95	48.01	49.88
	L115	L381	43.48	43.93	43.70	2.81	47.52	48.37	49.82
	L326	L381	42.34	43.93	43.30	2.93	47.15	48.10	49.73
	L37	L381	41.70	43.93	42.86	3.13	47.05	48.04	49.64
0	L329	L5	44.19	44.71	44.43	2.69	48.01	48.91	50.04
	L5	P085	44.71	42.79	43.71	2.80	47.37	48.24	49.81
	L157	L5	43.35	44.71	43.98	2.67	47.43	48.40	49.78
	L51	L5	42.55	44.71	43.62	2.89	47.55	48.46	49.75
	L43	L5	43.04	44.71	43.81	2.86	47.66	48.50	49.75
00	L367	L409	42.62	41.94	42.38	3.35	46.83	48.01	49.29
	L184	L311	42.68	44.00	43.37	2.23	46.31	47.18	48.48
	L311	L409	44.00	41.94	42.94	2.56	46.36	46.96	48.24
	L311	L367	44.00	42.62	43.29	2.49	46.70	47.30	48.20
	L380	L409	42.90	41.94	42.48	2.47	45.72	46.50	47.91
000	L181	L54	41.91	46.07	44.00	2.61	47.43	48.25	49.69
	L152	L54	44.09	46.07	45.08	2.31	48.20	48.72	49.55
	L247	L54	41.68	46.07	43.89	2.52	47.19	48.06	49.26
	L54	P055	46.07	41.88	43.93	2.59	47.33	48.11	49.18
	L289	L54	41.58	46.07	43.81	2.57	47.26	47.93	49.14
Entire	L54	L5	46.07	44.71	45.37	2.86	49.16	50.00	51.54
	L381	L54	43.93	46.07	45.04	2.82	48.71	49.63	51.14
	L326	L54	42.34	46.07	44.15	3.07	48.13	49.15	51.12
	L177	L54	44.34	46.07	44.66	2.76	48.34	49.31	50.75
	L37	L54	41.70	46.07	43.88	3.27	48.24	49.32	50.73

*P1 and P2 are the parents of a simple cross. Y1 and Y2, the means of observed SPC. P90, P95, and P99 indicate 90^th^, 95^th^, and 99^th^ percentile of the homozygous progeny value in the cross*.

### Annotation of Candidate Gene System of SPC in the NECSGP

Using the chi-square test, a total of 190 genes were significantly associated with 44 SPC QTLs in this study, and then 120 candidate genes on 34 SPC QTLs were annotated and functionally classified into 13 GO biological process categories, including transporter activity, translation, regulation of the biological process, metabolic process, transcription, phosphorylation, catabolic process, cellular process, response to stimulus, signaling, biosynthetic process, reproductive process, and others (Figure 1H). These candidate genes involved 34 SPC QTLs, explaining 41.35% of the PV (Supplementary Table 4). Among the candidate genes, four are involved directly in protein or amino acid synthesis and metabolism, according to the annotation information. The *Glyma03g33360* gene on *q-Prot-3-5* is involved in the histidine biosynthetic process. In NECSGP, six SNPs related to this gene were found, among which three SNPs were located within the gene and three SNPs were located in the 5 kb upstream and downstream of the gene. Significant differences in SPC were observed among the five haplotypes on this gene locus. The haplotype “AACTTC” had the highest frequency but lowest mean SPC in the NECSGP (Supplementary Figure 1A). The *Glyma15g10780* gene on *q-Prot-15-2* was involved in the S-adenosylmethioninamine biosynthetic process and the *Glyma16g29760* gene on *q-Prot-16-3* was involved in the peptidyl-pyroglutamic acid biosynthetic process. In these two loci, each contained only one SNP in NECSGP, and no significant associations between the SNP and SPC were observed (Supplementary Figures 1B,C). The *Glyma17g35490* gene on *q-Prot-17-6* involved in proteolysis, and its homologous gene in *Arabidopsis thaliana, AT5G67360*, belongs to the subtilase family protein, encoding a subtilisin-like serine protease essential for mucilage release from seed coats. The *Glyma17g35490* gene locus had seven haplotypes in NECSGP, and there were significant differences in SPC among haplotypes. The haplotype “GACTA” had the highest mean SPC while “GCACA” had the highest frequency in NECSGP (Supplementary Figure 1D). The above candidate gene information was cited and inferred from the SoyBase (http://soybase.org), and the biological functions of the candidate genes are to be studied and confirmed further. This information implied that SPC is a complex trait conferred by a gene network involving a series of functional genes.

## Discussion

### Genetic Potential and Optimal Cross Design of SPC in the NECSGP

The SPC in NECSGP varied greatly but was not as wide as that in the Chinese soybean landrace population. A slightly significant increase was observed from the old MGs to the new MGs. Using RTM-GWAS, 61 main-effect SPC QTLs with 240 alleles were detected, explaining 62.72% of the phenotypic variation. Based on the SPC QTL matrix, the predicted 95th percentile of SPC in progenies of possible crosses showed that the mean recombination potential was estimated as 43.29 or 2.52% more than the population mean of 40.77%. The maximum recombination potential was 50.00 or 9.23% more than the population means, and the maximum transgressive potential was 50.00 or 3.93% more than the best accession in the population. Thus, there was large genetic potential in improving SPC even though the phenotypic variation was not large in the population, and the genetic potential was mainly due to allele recombination in the population. Since both the linkage model and independent models had similar estimates in the prediction of recombination potential, there was no need to break linkage drags to improve SPC in the NECSGP. This result might apply to the soybeans in the Americas because the germplasm in the Americas was mainly introduced from the NECSGP. Of course, in addition to utilizing the recombination potential in the NECSGP itself, there should be more potential for a breakthrough in the improvement of SPC, if elite SPC germplasm is introduced to the NECSGP from external genetic resources.

Based on the above estimation of genetic recombination potential in the NECSGP, the optimal crosses were selected for breeding purposes. In other words, the present study has provided an optimal cross design procedure for SPC improvement, including the following steps: the establishment of a QTL-allele matrix based on RTM-GWAS, then simulation of the possible crosses done *in silico* for their breeding values of certain (95th for example) percentile homogeneous progenies, and finally choosing the best crosses according to the predicted breeding values. In this way, the best crosses or the best parental combinations are designed. Compared to the traditional breeding, this optimal cross design procedure covers all possible crosses in the population based on the establishment of a whole-genome QTL-allele matrix and is effective and efficient in predicting best crosses and progenies, realizing transformation from phenotype selection to genomic selection and shortening the breeding cycles. In addition, among the present possible crosses, 1,803 transgressive combinations were detected, in which the predicted best cross was L54 × L59 with SPC 50.00% in its 95th percentile progeny. Of the 73 SPC QTLs in these two parents, 42 had the same alleles and 31 had different alleles. Both parents had complementary large positive and negative effect alleles. L54 had one favorable allele (1.84%) on *q-Prot-13-1* and one allele with a large negative effect (-1.30%) on *q-Prot-4-6*, while L5 had five favorable alleles (0.79-1.88%) on *q-Prot-1-4, q-Prot-4-6, q-Prot-9-2, q-Prot-17-5, q-Prot-18-3*, and three alleles of a large negative effect (-0.97-1.84%) on *q-Prot-6-4, q-Prot-13-1*, and *q-Prot-18-1*, respectively. This example explained why L54 × L59 was the best-predicted cross and why the NECSGP has potential for SPC improvement through genetic recombination.

The above optimal cross prediction procedure is, in fact, a genome-wide sequencing marker-assisted prediction. Our previous marker-assisted selection for transgressive SPC in recombinant inbred line (RIL) populations was very effective (Zhang et al., 2015a). Two transgressive segregants for SPC with SPC of 49.33% and 46.32% were selected from two RIL populations with their parental SPC of 44.83, 44.83, 35.35, and 44.34%, respectively, and then were crossed for further improvement of SPC. The two transgressive segregants and the derived offspring were genotyped at three major SPC QTLs, and the recombinants with all three alleles of positive effect performed the highest SPC in F_2_-derived families, especially in the F_2:5:6_ generation where a progeny with the highest SPC of 54.15% was obtained. This example demonstrated the effectiveness of the marker-assisted selection procedure in breeding for SPC. Thus, this should also apply to the above predicted optimal cross L54 × L59; especially, it was based on whole-genome sequencing marker-assisted prediction, while Zhang et al.'s example was based only on some SSR markers.

In the present study, SPC was the primary focus, but modern breeders have been pursuing high-yield, high SPC, and high oil content soybean cultivars (Patil et al., 2017). Previous studies have found that soybean protein content was negatively correlated with oil content and yield (Chaudhary et al., 2015). High protein content often leads to a decrease in oil content and yield. In breeding soybean cultivars with high protein content, high oil content, and high yield, balancing the relationship among the three traits has always been an urgent problem to be solved. In the present study, it is suggested to establish the QTL-allele matrices for all the three traits, on which the optimal crosses for combining all elite QTL-alleles of the three traits might be predicted. Therefore, optimal cross prediction for multiple traits should be further explored.

### The SPC QTL-Allele Structure and Evolutionary Mechanism in the NECSGP

In the NECSGP, 73 SPC QTLs/SNPLDBs with 273 alleles were detected, accounting for 71.70% PV, in which 61 main-effect QTLs with 240 alleles accounted for 62.72% PV. Compared to the QTL reported in the literature and SoyBase (https://soybase.org), 45 QTLs overlapped with the reported QTLs, and 28 QTLs were newly found, explaining 23.85% PV. The SNPLDB markers also satisfied the requirements of the presence of multiple alleles in natural populations. The QTL of the largest contribution was *q-Prot-4-6*, which explained 2.32% PV. Compared to previous studies (Bandillo et al., 2015; Sonah et al., 2015; Zhang et al., 2017), QTLs with relatively small effects could also be detected for SPC in this study using the RTM-GWAS method; in other words, the SPC QTLs with their alleles can be fully explored. By taking the trait heritability as the upper PV limit, both the false positive and false negative problems can be controlled in the RTM-GWAS method. The detection power was further boosted with the two-stage analysis strategy and the multi-locus model. Thus, the relatively thorough detection of the SPC QTL-allele system in the NECSGP can facilitate the study of genetic dynamics of SPC variation.

The SPC QTL-allele structure changed from the old MGs to the new MGs, with both emerged alleles and excluded alleles, but allele changes in SPC were not as many as those in days to flowering (Fu et al., 2020b; Liu et al., 2021), main stem node number (Fu et al., 2020a; Fahim et al., 2021), and other traits (Meng et al., 2016). Therefore, SPC is a trait not sensitive to allele changes, which may be one reason why SPC cannot be improved readily. However, among the four evolutionary motivators of allele inheritance, emergence, exclusion, and recombination, the allele contributions for the first three factors were 97.79%, 2.21%, and 0.75%, respectively. The allele emergence and allele exclusion were relatively weak in SPC. The fourth factor, allele recombination, was relatively strong as indicated in the prediction of recombination potential. Thus, for a breakthrough in improving SPC in the NECSGP, introducing superior alleles from other germplasm populations may be a potential strategy for SPC breeding in NEC.

Furthermore, 34 out of the 73 QTLs (SNPLDBs) had only two alleles, of which 31 QTLs were SNPLDBs containing only a single SNP (S.SNPLDB). Previous studies showed that along with the increase in the number of SNPs or sequencing depth, the S.SNPLDBs would likely be merged into LD blocks with multiple SNPs (He et al., 2017). Since the detected SNP number of the soybean genome in this study was relatively small, the exploration of SPC QTL-allele in the NECSGP may be further improved with sequencing depth increased.

## Conclusion

The SPC in NECSGP varied greatly but was not as high as in the Chinese soybean landrace population. There was a slight SPC increase from the old MGs (III + II + I) to the new MGs (0 + 00 + 000). The 71.70% SPC variation in NECSGP can be explained by 73 SPC QTLs with 273 alleles, including 28 newly identified QTLs. The evolutionary changes of QTL-allele structure from old MGs to new MGs showed most alleles in new MGs were inherited from the old MGs, and only a small number of alleles emerged or were excluded. The small amount of new positive allele emergence and possible allele recombination between alleles explained the slight SPC increase in new MGs. The prediction results of 95th percentile progenies of possible crosses showed recombination and transgressive potential, indicating that SPC breeding potentials exist in NECSGP. Candidate gene analysis indicated that SPC is a complex trait conferred by a gene network involving a series of functional genes.

## Data Availability Statement

The datasets presented in this study can be found in online repositories. The names of the repository/repositories and accession number(s) can be found below: https://github.com/njau-sri/NECSGP-SPC, NECSGP-SPC.

## Author Contributions

JG designed the experiments. LF, MF, YW, HR, and WD performed the field experiments. WF, ZS, and JH analyzed the data and interpreted the results. XH and JZ participated in data analysis. LS, LW, WW, and GX participated in field experiments. WF, JH, ZS, and JG drafted the manuscript. All authors approved the manuscript.

## Funding

This work was supported by the National Key Research Development Program of China (2021YFF1001204), the Program of Jiangsu Province (JBGS-2021-014), the MOE 111 Project (B08025), the MOE Program for Changjiang Scholars and Innovative Research Team in University (PCSIRT_17R55), the Fundamental Research Funds for the Central Universities (KYZZ201901), the MARA CARS-04 Program, the Primary Research and Development Plan of Jiangsu Province (BE2021358), the Jiangsu JCIC-MCP, the Guidance Foundation of Sanya Institute of Nanjing Agricultural University (NAUSY-ZZ02 and NAUSY-MS05), and the Bioinformatics Center of Nanjing Agricultural University.

## Conflict of Interest

The authors declare that the research was conducted in the absence of any commercial or financial relationships that could be construed as a potential conflict of interest.

## Publisher's Note

All claims expressed in this article are solely those of the authors and do not necessarily represent those of their affiliated organizations, or those of the publisher, the editors and the reviewers. Any product that may be evaluated in this article, or claim that may be made by its manufacturer, is not guaranteed or endorsed by the publisher.
