# Supplementary material for: Transgressive Potential Prediction and Optimal Cross Design of Seed Protein Content in the Northeast China Soybean Population Based on Full Exploration of the QTL-Allele System
Source: Front Plant Sci. 2022 Jul 12;13:896549. doi: 10.3389/fpls.2022.896549 (PMC9317943; doi:10.3389/fpls.2022.896549)
Supplement: Supplementary file 1 [file Table_1.DOCX]

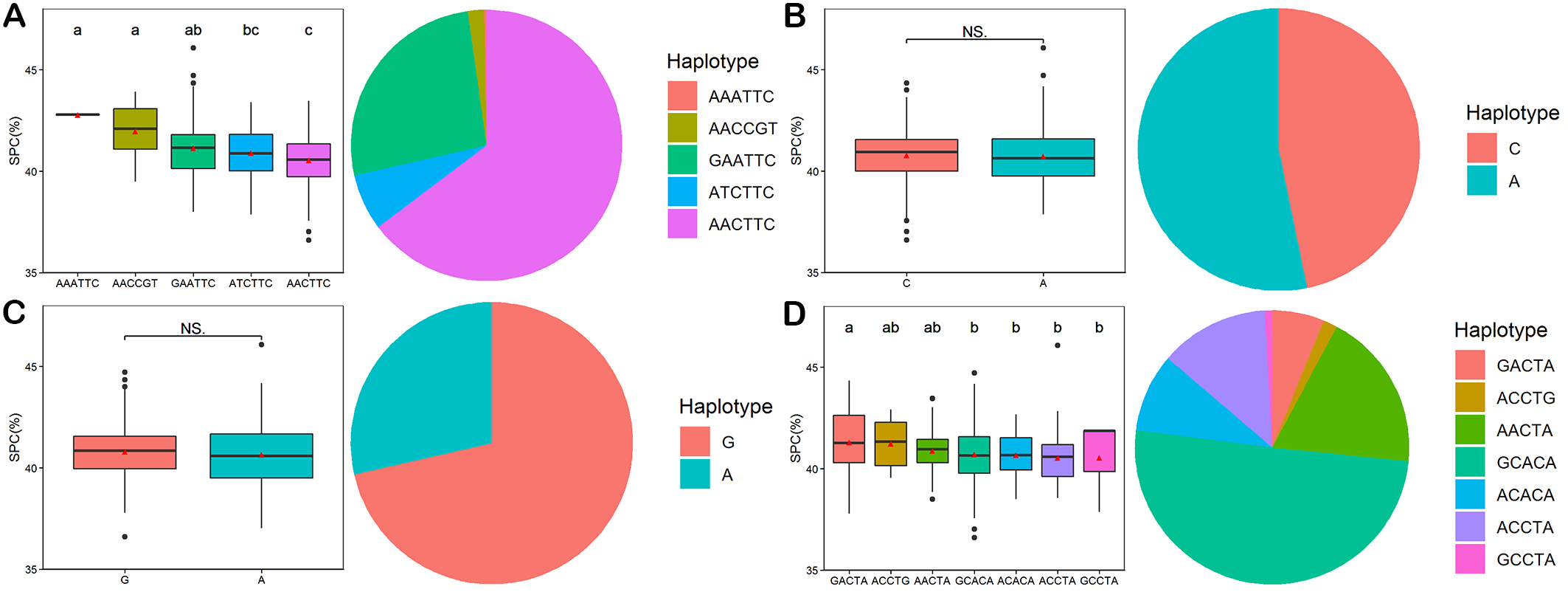


**Supplementary Figure 1** The genotypic effect and frequency of haplotype of candidate genes. **A**, *Glyma03g33360*; **B**, *Glyma15g10780*; **C**, *Glyma16g29760*; **D**, *Glyma17g35490*. The left part: the SPC of haplotype of candidate genes, The right part: the frequency of haplotype of candidate genes. The red triangle represents the average haplotype phenotype. NS. indicates no significant difference. Multiple comparisons are represented by the lowercase letter ‘a’, ‘b’, ‘c’ and the significance level is 0.05.
