# Supplementary material for: Transgressive Potential Prediction and Optimal Cross Design of Seed Protein Content in the Northeast China Soybean Population Based on Full Exploration of the QTL-Allele System
Source: Front Plant Sci. 2022 Jul 12;13:896549. doi: 10.3389/fpls.2022.896549 (PMC9317943; doi:10.3389/fpls.2022.896549)
Supplement: Supplementary file 2 [file Data_Sheet_1.docx]

**Supplementary Tables**

**Supplementary Table 1｜**Joint analysis of variance for SPC in Northeast China soybean germplasm population

| Source of variation | DF | MS | *F* |
| --- | --- | --- | --- |
| Env. | 1 | 1034.61 | 143.43** |
| Block (Env.) | 6 | 5.54 | 6.96** |
| Accession | 360 | 14.69 | 5.88** |
| Accession×Env. | 356 | 2.50 | 3.14** |
| Error | 2129 | 0.80 |  |
| Total | 2852 |  |  |

**significant difference at level of *P* < 0.01.

**Supplementary Table 2｜**The allele effects of detected SPC QTLs in Northeast China soybean germplasm population

| QTL | AN | a1 | a2 | a3 | a4 | a5 | a6 | a7 | a8 | a9 | a10 |
| --- | --- | --- | --- | --- | --- | --- | --- | --- | --- | --- | --- |
| *q-Prot-1-1* | 2 | -0.23 | 0.23 |  |  |  |  |  |  |  |  |
| *q-Prot-1-2* | 2 | -0.47 | 0.47 |  |  |  |  |  |  |  |  |
| *q-Prot-1-3* | 2 | -0.01 | 0.01 |  |  |  |  |  |  |  |  |
| *q-Prot-1-4* | 5 | -0.50 | -0.24 | -0.04 | -0.02 | 0.79 |  |  |  |  |  |
| *q-Prot-2-1* | 6 | -0.88 | -0.37 | -0.24 | -0.22 | -0.04 | 1.75 |  |  |  |  |
| *q-Prot-3-1* | 6 | -1.41 | -0.10 | 0.15 | 0.33 | 0.44 | 0.60 |  |  |  |  |
| *q-Prot-3-2* | 3 | -0.17 | -0.15 | 0.32 |  |  |  |  |  |  |  |
| *q-Prot-3-3* | 8 | -0.85 | -0.66 | -0.61 | -0.36 | -0.13 | 0.28 | 0.87 | 1.46 |  |  |
| *q-Prot-3-4* | 2 | -0.85 | 0.85 |  |  |  |  |  |  |  |  |
| *q-Prot-3-5* | 8 | -1.42 | -1.12 | -0.01 | 0.15 | 0.29 | 0.44 | 0.46 | 1.21 |  |  |
| *q-Prot-3-6* | 2 | -0.43 | 0.43 |  |  |  |  |  |  |  |  |
| *q-Prot-3-7* | 6 | -1.18 | -0.27 | 0.26 | 0.30 | 0.38 | 0.51 |  |  |  |  |
| *q-Prot-4-1* | 10 | -0.39 | -0.33 | -0.09 | -0.09 | 0.00 | 0.08 | 0.09 | 0.11 | 0.17 | 0.45 |
| *q-Prot-4-2* | 2 | -0.27 | 0.27 |  |  |  |  |  |  |  |  |
| *q-Prot-4-3* | 8 | -1.18 | -0.38 | -0.03 | 0.06 | 0.14 | 0.24 | 0.55 | 0.60 |  |  |
| *q-Prot-4-4* | 2 | -1.40 | 1.40 |  |  |  |  |  |  |  |  |
| *q-Prot-4-5* | 2 | -0.36 | 0.36 |  |  |  |  |  |  |  |  |
| *q-Prot-4-6* | 4 | -1.30 | -0.14 | 0.51 | 0.93 |  |  |  |  |  |  |
| *q-Prot-5-1* | 2 | -0.17 | 0.17 |  |  |  |  |  |  |  |  |
| *q-Prot-5-2* | 6 | -0.38 | -0.30 | -0.18 | 0.01 | 0.41 | 0.44 |  |  |  |  |
| *q-Prot-6-1* | 4 | -0.87 | -0.14 | -0.06 | 1.07 |  |  |  |  |  |  |
| *q-Prot-6-2* | 2 | -0.19 | 0.19 |  |  |  |  |  |  |  |  |
| *q-Prot-6-3* | 2 | -0.34 | 0.34 |  |  |  |  |  |  |  |  |
| *q-Prot-6-4* | 3 | -1.35 | -0.46 | 1.81 |  |  |  |  |  |  |  |
| *q-Prot-7-1* | 2 | -0.73 | 0.73 |  |  |  |  |  |  |  |  |
| *q-Prot-7-2* | 4 | -0.38 | -0.06 | 0.07 | 0.37 |  |  |  |  |  |  |
| *q-Prot-8-1* | 3 | -0.22 | -0.09 | 0.31 |  |  |  |  |  |  |  |
| *q-Prot-8-2* | 2 | -0.25 | 0.25 |  |  |  |  |  |  |  |  |
| *q-Prot-8-3* | 8 | -1.89 | -0.15 | -0.08 | 0.11 | 0.16 | 0.26 | 0.43 | 1.16 |  |  |
| *q-Prot-8-4* | 2 | -0.36 | 0.36 |  |  |  |  |  |  |  |  |
| *q-Prot-9-1* | 5 | -0.59 | -0.34 | 0.17 | 0.30 | 0.47 |  |  |  |  |  |
| *q-Prot-9-2* | 4 | -1.42 | -0.25 | 0.11 | 1.57 |  |  |  |  |  |  |
| *q-Prot-9-3* | 4 | -1.15 | 0.01 | 0.10 | 1.04 |  |  |  |  |  |  |
| *q-Prot-9-4* | 2 | -0.51 | 0.51 |  |  |  |  |  |  |  |  |
| *q-Prot-9-5* | 5 | -0.40 | -0.36 | 0.09 | 0.33 | 0.35 |  |  |  |  |  |
| *q-Prot-10-1* | 4 | -1.25 | 0.19 | 0.50 | 0.56 |  |  |  |  |  |  |
| *q-Prot-10-2* | 2 | -0.17 | 0.17 |  |  |  |  |  |  |  |  |
| *q-Prot-10-3* | 2 | -0.20 | 0.20 |  |  |  |  |  |  |  |  |
| *q-Prot-11-1* | 2 | -0.12 | 0.12 |  |  |  |  |  |  |  |  |
| *q-Prot-12-1* | 4 | -1.05 | 0.25 | 0.38 | 0.42 |  |  |  |  |  |  |
| *q-Prot-12-2* | 2 | -0.15 | 0.15 |  |  |  |  |  |  |  |  |
| *q-Prot-12-3* | 2 | -1.01 | 1.01 |  |  |  |  |  |  |  |  |
| *q-Prot-13-1* | 2 | -1.84 | 1.84 |  |  |  |  |  |  |  |  |
| *q-Prot-13-2* | 5 | -0.54 | -0.11 | -0.09 | -0.09 | 0.83 |  |  |  |  |  |
| *q-Prot-14-1* | 3 | -0.27 | 0.04 | 0.23 |  |  |  |  |  |  |  |
| *q-Prot-14-2* | 2 | -0.11 | 0.11 |  |  |  |  |  |  |  |  |
| *q-Prot-14-3* | 2 | -0.43 | 0.43 |  |  |  |  |  |  |  |  |
| *q-Prot-15-1* | 2 | -0.45 | 0.45 |  |  |  |  |  |  |  |  |
| *q-Prot-15-2* | 6 | -0.64 | -0.36 | -0.33 | -0.25 | 0.57 | 1.01 |  |  |  |  |
| *q-Prot-15-3* | 3 | -0.60 | 0.04 | 0.56 |  |  |  |  |  |  |  |
| *q-Prot-15-4* | 2 | 0.11 | -0.11 |  |  |  |  |  |  |  |  |
| *q-Prot-16-1* | 4 | -0.13 | -0.02 | 0.06 | 0.09 |  |  |  |  |  |  |
| *q-Prot-16-2* | 2 | -0.45 | 0.45 |  |  |  |  |  |  |  |  |
| *q-Prot-16-3* | 4 | -0.71 | -0.35 | -0.12 | 1.18 |  |  |  |  |  |  |
| *q-Prot-17-1* | 4 | -0.99 | 0.07 | 0.37 | 0.55 |  |  |  |  |  |  |
| *q-Prot-17-2* | 4 | -0.37 | -0.02 | 0.19 | 0.20 |  |  |  |  |  |  |
| *q-Prot-17-3* | 6 | -0.47 | -0.28 | -0.27 | -0.04 | -0.04 | 1.10 |  |  |  |  |
| *q-Prot-17-4* | 2 | -1.01 | 1.01 |  |  |  |  |  |  |  |  |
| *q-Prot-17-5* | 6 | -1.83 | -0.23 | -0.04 | 0.44 | 0.83 | 0.83 |  |  |  |  |
| *q-Prot-17-6* | 7 | -0.49 | -0.30 | -0.25 | -0.16 | -0.06 | -0.04 | 1.30 |  |  |  |
| *q-Prot-18-1* | 9 | -1.56 | -1.05 | -0.97 | -0.10 | -0.07 | -0.05 | 0.55 | 1.40 | 1.85 |  |
| *q-Prot-18-2* | 2 | -0.39 | 0.39 |  |  |  |  |  |  |  |  |
| *q-Prot-18-3* | 5 | -1.87 | -0.86 | -0.66 | 1.52 | 1.88 |  |  |  |  |  |
| *q-Prot-19-1* | 2 | -0.29 | 0.29 |  |  |  |  |  |  |  |  |
| *q-Prot-19-2* | 7 | -0.73 | -0.53 | -0.29 | -0.11 | 0.19 | 0.29 | 1.18 |  |  |  |
| *q-Prot-19-3* | 2 | -0.45 | 0.45 |  |  |  |  |  |  |  |  |
| *q-Prot-19-4* | 4 | -0.68 | -0.60 | -0.30 | 1.58 |  |  |  |  |  |  |
| *q-Prot-20-1* | 2 | -0.49 | 0.49 |  |  |  |  |  |  |  |  |
| *q-Prot-20-2* | 3 | -1.14 | 0.55 | 0.59 |  |  |  |  |  |  |  |
| *q-Prot-20-3* | 7 | -0.56 | -0.40 | -0.34 | -0.30 | 0.22 | 0.62 | 0.76 |  |  |  |
| *q-Prot-20-4* | 2 | -0.75 | 0.75 |  |  |  |  |  |  |  |  |
| *q-Prot-20-5* | 2 | -0.20 | 0.20 |  |  |  |  |  |  |  |  |
| *q-Prot-20-6* | 2 | -0.41 | 0.41 |  |  |  |  |  |  |  |  |

AN, the number of alleles; a1, a2, …, a10, the allele codes for each QTL.

**Supplementary Table 3｜**A list of overlapped loci with their position reported in SoyBase

| QTL | Chr. | Position(bp) | SNPs ^a^ | SoyBase QTL |
| --- | --- | --- | --- | --- |
| *q-Prot-1-1* | 1 | 7051961 | 1 | 3-4, 3-5 |
| *q-Prot-1-2* | 1 | 17175223 | 1 | 3-4 |
| *q-Prot-1-3* | 1 | 22244385 | 1 | 3-4 |
| *q-Prot-1-4* | 1 | 47943457-47993349 | 11 | 3-4, 40-4 |
| *q-Prot-2-1* | 2 | 3224504-3386591 | 10 |  |
| *q-Prot-3-1* | 3 | 9883450-10082878 | 15 |  |
| *q-Prot-3-2* | 3 | 20016925-20165235 | 9 |  |
| *q-Prot-3-3* | 3 | 27140827-27314739 | 18 |  |
| *q-Prot-3-4* | 3 | 28211045 | 1 |  |
| *q-Prot-3-5* | 3 | 41008189-41201610 | 20 | 21-9, 36-34 |
| *q-Prot-3-6* | 3 | 43342844 | 1 | 27-4, 36-34, 36-35 |
| *q-Prot-3-7* | 3 | 45585122-45784092 | 20 | 27-4 |
| *q-Prot-4-1* | 4 | 15539474-15739342 | 37 | 19-1 |
| *q-Prot-4-2* | 4 | 19645086 | 1 | 19-1 |
| *q-Prot-4-3* | 4 | 31245142-31363647 | 35 |  |
| *q-Prot-4-4* | 4 | 34306302 | 1 |  |
| *q-Prot-4-5* | 4 | 34388114 | 1 |  |
| *q-Prot-4-6* | 4 | 36804595-36956694 | 24 |  |
| *q-Prot-5-1* | 5 | 15487830 | 1 |  |
| *q-Prot-5-2* | 5 | 33803074-33915070 | 23 |  |
| *q-Prot-6-1* | 6 | 6871018-6911128 | 6 | 30-5 |
| *q-Prot-6-2* | 6 | 12122955 | 1 |  |
| *q-Prot-6-3* | 6 | 29254180 | 1 | 28-1, 29-1, 35-2, 36-7, 36-8 |
| *q-Prot-6-4* | 6 | 31465819-31481467 | 7 | 28-1, 29-1, 35-2, 36-7, 36-8 |
| *q-Prot-7-1* | 7 | 21000729 | 1 | 41-9 |
| *q-Prot-7-2* | 7 | 36859343-36901664 | 4 | 41-9 |
| *q-Prot-8-1* | 8 | 5223834-5308407 | 5 |  |
| *q-Prot-8-2* | 8 | 25835448 | 1 |  |
| *q-Prot-8-3* | 8 | 30320715-30453625 | 26 |  |
| *q-Prot-8-4* | 8 | 34516909 | 1 |  |
| *q-Prot-9-1* | 9 | 15918825-16040813 | 13 | 36-28, 36-29, 37-10, 47-2 |
| *q-Prot-9-2* | 9 | 17559197-17575587 | 5 | 36-28, 36-29, 37-10, 47-2 |
| *q-Prot-9-3* | 9 | 28009070-28068638 | 5 | 36-28, 36-29, 36-30, 37-10, 47-2 |
| *q-Prot-9-4* | 9 | 29398432-29398594 | 2 | 36-28, 36-29, 36-30, 37-10, 47-2 |
| *q-Prot-9-5* | 9 | 34748141-34946569 | 45 | 5-3, 30-8, 35-4, 36-28, 36-29, 36-30, 37-10 |
| *q-Prot-10-1* | 10 | 7564092-7666635 | 17 | 41-11 |
| *q-Prot-10-2* | 10 | 34620415 | 1 | 36-40 |
| *q-Prot-10-3* | 10 | 45609679 | 1 |  |
| *q-Prot-11-1* | 11 | 36522210 | 1 |  |
| *q-Prot-12-1* | 12 | 10419180-10443940 | 20 | 34-8, 47-3 |
| *q-Prot-12-2* | 12 | 21248348-21248477 | 2 |  |
| *q-Prot-12-3* | 12 | 34526609 | 1 |  |
| *q-Prot-13-1* | 13 | 22819454 | 1 | 3-7, 36-20, 36-21, 36-23 |
| *q-Prot-13-2* | 13 | 35240772-35295737 | 5 | 24-2, 36-20, 36-21 |
| *q-Prot-14-1* | 14 | 1631406-1664408 | 4 |  |
| *q-Prot-14-2* | 14 | 23588110 | 1 | 4-10 |
| *q-Prot-14-3* | 14 | 28584585 | 1 | 4-10 |
| *q-Prot-15-1* | 15 | 6935-6981 | 2 |  |
| *q-Prot-15-2* | 15 | 7483684-7680382 | 11 | 3-6, 5-1, 39-2 |
| *q-Prot-15-3* | 15 | 40991110-40991116 | 2 |  |
| *q-Prot-15-4* | 15 | 41520869 | 1 |  |
| *q-Prot-16-1* | 16 | 8004288-8203845 | 12 |  |
| *q-Prot-16-2* | 16 | 31362755 | 1 |  |
| *q-Prot-16-3* | 16 | 33388199-33538361 | 6 |  |
| *q-Prot-17-1* | 17 | 14577507-14601127 | 16 | 26-2, 36-17 |
| *q-Prot-17-2* | 17 | 15717972-15782305 | 11 | 26-2, 36-17 |
| *q-Prot-17-3* | 17 | 21969506-22037018 | 17 | 36-15, 36-17, 47-5 |
| *q-Prot-17-4* | 17 | 34798780 | 1 | 36-14, 36-16 |
| *q-Prot-17-5* | 17 | 35769952-35842671 | 9 | 36-14, 36-16 |
| *q-Prot-17-6* | 17 | 39638460-39801567 | 13 | 30-2, 39-3 |
| *q-Prot-18-1* | 18 | 7317014-7504444 | 32 | 36-25, 47-7 |
| *q-Prot-18-2* | 18 | 27467805 | 1 | 36-25, 47-7 |
| *q-Prot-18-3* | 18 | 58197364-58341700 | 14 | 3-10, 36-25 |
| *q-Prot-19-1* | 19 | 6721655 | 1 | 33-4 |
| *q-Prot-19-2* | 19 | 21347881-21386285 | 15 | 34-10 |
| *q-Prot-19-3* | 19 | 21466495 | 1 | 34-10 |
| *q-Prot-19-4* | 19 | 47660663-47715521 | 16 |  |
| *q-Prot-20-1* | 20 | 2795994 | 1 | 1-3, 1-4, 3-12, 11-1, 30-1, 36-26, 47-8 |
| *q-Prot-20-2* | 20 | 14150016-14150305 | 3 | 1-3, 1-4, 3-12, 10-1, 11-1, 30-1, 31-1, 36-26, 37-8, 47-8 |
| *q-Prot-20-3* | 20 | 15240451-15421786 | 7 | 1-3, 1-4, 3-12, 10-1, 11-1, 30-1, 31-1, 36-26, 37-8, 47-8 |
| *q-Prot-20-4* | 20 | 31571297 | 1 | 1-1, 1-2, 15-1, 30-1, 31-1, 34-11, 36-26, 37-8, 39-4 |
| *q-Prot-20-5* | 20 | 33063637 | 1 | 1-1, 1-2, 26-5, 30-1, 34-11, 36-26 |
| *q-Prot-20-6* | 20 | 42200759 | 1 | 35-3 |

Chr., chromosome.

^a^ the number of SNP markers within the SNPLDB marker.

**Supplementary Table 4｜**Functional annotation of candidate genes for SPC QTLs in the NECSGP

| QTL | *R^2^*(%) | Gene ^a^ |
| --- | --- | --- |
| *q-Prot-1-4* | 0.97 | *Glyma01g35480* (8) |
| *q-Prot-2-1* | 2.24 | *Glyma02g03800* (1,8,12); *Glyma02g03920* (7); *Glyma02g04010* (6); *Glyma02g04070* (2); *Glyma02g04110* (2); *Glyma02g04190* (3); *Glyma02g04360* (4); *Glyma02g04381* (1,7) |
| *q-Prot-3-3* | 1.97 | *Glyma03g21650* (1) |
| *q-Prot-3-5* | 0.74 | *Glyma03g33160* (1,8); *Glyma03g33240* (1,8); ***Glyma03g33360*** (11); *Glyma03g33460* (2); *Glyma03g33470* (3,5); *Glyma03g33680* (1,8); *Glyma03g33850* (1,10) |
| *q-Prot-3-6* | 1.02 | *Glyma03g36130* (5); *Glyma03g36240* (6,10); *Glyma03g36450* (2) |
| *q-Prot-3-7* | 3.03 | *Glyma03g39530* (8); *Glyma03g39570* (2,7); *Glyma03g39600* (1); *Glyma03g39730* (4,7,11); *Glyma03g39760* (6,10,12); *Glyma03g39850* (1) |
| *q-Prot-4-1* | 0.43 | *Glyma04g14970* (1,3); *Glyma04g15093* (9); *Glyma04g15130* (2); *Glyma04g15160* (1) |
| *q-Prot-4-6* | 2.32 | *Glyma04g32217* (9,13); *Glyma04g32301* (2) |
| *q-Prot-5-2* | 0.49 | *Glyma05g28070* (4,6,9,10,12) |
| *q-Prot-6-1* | 1.35 | *Glyma06g09220* (4); *Glyma06g09310* (5,8); *Glyma06g09320* (4,6,7); *Glyma06g09345* (3,6,13); *Glyma06g09340* (3,6,13) |
| *q-Prot-6-2* | 1.15 | *Glyma06g15370* (5) |
| *q-Prot-7-2* | 0.53 | *Glyma07g31870* (3); *Glyma07g31995* (3,5); *Glyma07g32050* (2,9); *Glyma07g32110* (2,9) |
| *q-Prot-8-1* | 0.22 | *Glyma08g07001* (3,5); *Glyma08g07050* (6,9,10); *Glyma08g07110* (4,7); *Glyma08g07140* (4); *Glyma08g07260* (3,5,12); *Glyma08g07330* (2,9); *Glyma08g07400* (4); *Glyma08g07620* (7) |
| *q-Prot-9-1* | 0.53 | *Glyma09g14090* (6,10) |
| *q-Prot-9-2* | 1.85 | *Glyma09g15135* (12) |
| *q-Prot-9-5* | 0.71 | *Glyma09g27720* (6,9); *Glyma09g27940* (1); *Glyma09g27980* (5,12); *Glyma09g28030* (5) |
| *q-Prot-10-1* | 0.65 | *Glyma13g33390* (8); *Glyma13g33431* (5); *Glyma13g33450* (1); *Glyma13g33460* (2,3,4); *Glyma13g33730* (4,11) |
| *q-Prot-10-3* | 0.18 | *Glyma10g08710* (2,10); *Glyma10g08801* (8) |
| *q-Prot-13-2* | 0.18 | *Glyma10g37675* (4,5,12) |
| *q-Prot-14-1* | 0.20 | *Glyma14g02380* (7,11); *Glyma14g02450* (5); *Glyma14g02470* (7); *Glyma14g02530* (7); *Glyma14g02561* (3,13); *Glyma14g02780* (2,3,5); *Glyma14g02790* (6); *Glyma14g02930* (1) |
| *q-Prot-15-2* | 1.64 | *Glyma15g10210* (2,5); *Glyma15g10420* (5); *Glyma15g10520* (4,11); *Glyma15g10550* (6,13); *Glyma15g10700* (1); ***Glyma15g10780*** (11); *Glyma15g10791* (7) |
| *q-Prot-15-3* | 0.32 | *Glyma15g3593*1 (4) |
| *q-Prot-16-1* | 0.39 | *Glyma16g08430* (1,12); *Glyma16g08480* (1) |
| *q-Prot-16-2* | 0.24 | *Glyma16g27350* (1,4) |
| *q-Prot-16-3* | 1.17 | *Glyma16g29580* (1); ***Glyma16g29760*** (11); *Glyma16g29900* (3); *Glyma16g29910* (1,7); *Glyma16g29990* (1) |
| *q-Prot-17-1* | 0.60 | *Glyma17g17665* (3); *Glyma17g17710* (3) |
| *q-Prot-17-2* | 3.36 | *Glyma17g18350* (6); *Glyma17g18380* (2,7); *Glyma17g18450* (4,8,11) |
| *q-Prot-17-6* | 0.46 | *Glyma17g35490* (7,9); *Glyma17g35620* (3); *Glyma17g35650* (2,7); *Glyma17g35681* (5,12); ***Glyma17g35690*** (2,7); *Glyma17g35930* (3); *Glyma17g35960* (2); *Glyma17g36000* (9) |
| *q-Prot-18-1* | 1.42 | *Glyma18g08440* (6,9,10) |
| *q-Prot-18-3* | 2.74 | *Glyma18g48650* (4); *Glyma18g48700* (12); *Glyma18g48710* (10); *Glyma18g48930* (6,10); *Glyma18g49000* (4,6,11); *Glyma18g49020* (4,8); *Glyma18g49110* (1,8) |
| *q-Prot-19-4* | 4.86 | *Glyma19g41150* (5); *Glyma19g41371* (4); *Glyma19g41590* (3,8); *Glyma19g41651* (2); *Glyma19g41680* (5) |
| *q-Prot-20-1* | 1.39 | *Glyma20g03040* (11) |
| *q-Prot-20-3* | 1.16 | *Glyma20g10960* (3,6) |
| *q-Prot-20-6* | 0.84 | *Glyma20g33430* (3); *Glyma20g33491* (2); *Glyma20g33680* (8) |

^a^ The numbers in parenthesis indicate GO biological process classification. 1, transporter activity. 2, translation. 3, regulation of biological process. 4, metabolic process. 5, transcription. 6, phosphorylation. 7, catabolic process. 8, cellular process. 9, response to stimulus. 10, signaling. 11, biosynthetic process. 12, reproductive process. 13, other GO biological processes including snoRNA localization, localization, anatomical structure development, post-embryonic development, multicellular organism development, activation of protein kinase activity, Golgi organization, chloroplast organization, macromolecule methylation and methylation.
